# Modification of pre-operative order set to reduce PACU stay times for outpatient benign gynecological surgery

**DOI:** 10.1371/journal.pone.0336194

**Published:** 2026-06-10

**Authors:** Jessica Ainooson, Marcus Alonso Cee Williams, Rulan Yi, Heather Hervey-Jumper, Lee-lynn Chen, Stephanie Lim

**Affiliations:** 1 School of Medicine, University of California, San Francisco, California, United States of America; 2 Department of Bioengineering, University of California, San Francisco-Berkeley, California, United States of America; 3 Department of Anesthesia, University of California, San Francisco, California, United States of America; Monash University, INDONESIA

## Abstract

A quality improvement project was implemented at a single medical center’s outpatient benign gynecological surgery division to address prolonged post anesthesia care unit (PACU) stays (defined as greater than 120 minutes). Initial barrier analysis within the department identified gabapentin use in the perioperative setting as a possible contributor. The intervention removed the default pre-op order set of 600 mg gabapentin. One-year post-intervention (baseline n = 281, intervention n = 573) it was observed that the average PACU stay time for benign gynecological surgeries was reduced from an average of ~183 to ~159 minutes marking a 12.6% reduction in PACU stay time (p < 0.0001). Gabapentin use decreased from 92.17% to 1.25% with no change in pain scores. After the intervention, there was no longer a statistically significant difference in PACU stay times by age. This intervention successfully decreased PACU stay times, highlighting pathways to advance individualized, age-conscious care through quality improvement interventions.

## Introduction

Advances in laparoscopic surgery have expanded the opportunities for minimally invasive surgery, particularly in the field of gynecology. Today, minimally invasive gynecologic surgery (MIGS) is the standard of care for the treatment of many gynecological conditions including endometriosis, fibrosis, and ovarian cysts amongst others [1]. Outpatient benign gynecological surgery categories are among the top ambulatory procedures performed for females aged 18–64 years [2]. However, the rise of MIGS also highlights the challenge of safe and effective discharge from post anesthesia care unit (PACU). Many factors come into play from Nursing, Pharmacy, Anesthesia, and Surgery that contribute to the overall experience of the patient.

The PACU provides care to immediate postsurgical patients, occupying a key transition of care from the operating room to home. PACU stay times are impacted by multiple factors including pain, nausea, delay in orders, and patient communication [3]. Prolonged PACU stay for outpatient procedures, defined as greater than 120 minutes, can create further obstacles for patients or caregivers, and more stress and financial burden in the hospital, affecting overall PACU and operating room (OR) capacity [4]. Studying patient experience and identifying risk factors for prolonged (PACU) post anesthesia care unit stay can optimize resources used during care and the order of surgical cases in each day.

At this medical center, a tertiary care teaching hospital, the study team noted the outpatient benign gynecological (benign gyn) surgery PACU stays averaged 183 minutes in 2021, and in the past three years (2019–2021), this department has had the longest PACU stays of all surgical departments. Reducing the PACU stay time would improve patient-centered care and overall hospital performance. A quality improvement (QI) intervention was implemented by the study team as an effort to reduce PACU stay time for outpatient procedures in benign gyn through identifying and modifying health system factors.

## Methods

### Developing project goals

The identified clinical site was selected based on operational challenges related to PACU bed availability. A retrospective review of electronic health records (Epic) conducted for the years 2019–2021 to assess PACU stay durations across surgical departments revealed prolonged PACU stay times for outpatient benign gynecologic surgeries.

To understand barriers to timely PACU discharge in this department, a Qualtrics survey was developed and distributed to clinical staff. A total of 91 responses were received, including 27 registered nurses (RNs), 14 OB/GYN residents, 8 anesthesia residents, 6 OB/GYN attendings, 18 anesthesia attendings, 16 certified registered nurse anesthetists (CRNAs), 1 OB/GYN fellow, and 1 urogynecology attending. In addition, a multidisciplinary meeting was held with the benign gynecologic surgery team to review preliminary findings and inform subsequent quality improvement efforts.

**Fig 1:**
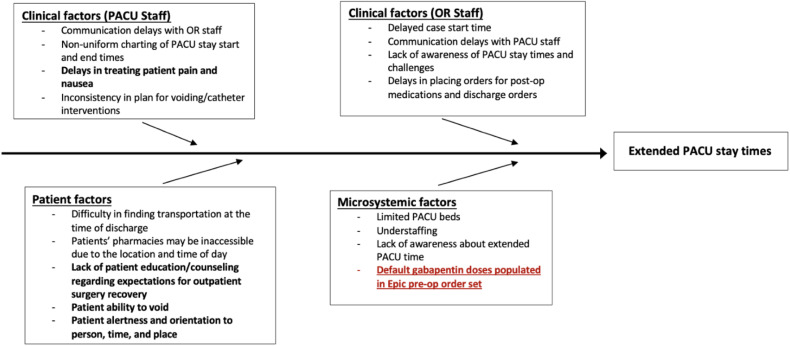
Fishbone diagram portraying perceived barrier to extended PACU stay times in outpatient benign gynecological surgeries.

To systematically identify and prioritize contributors to delayed PACU discharge, the study team first developed a fishbone diagram to categorize potential causes across domains such as clinical factors, medication protocols, patient factors, workflow, and communication (Fig 1).

**Fig 2:**
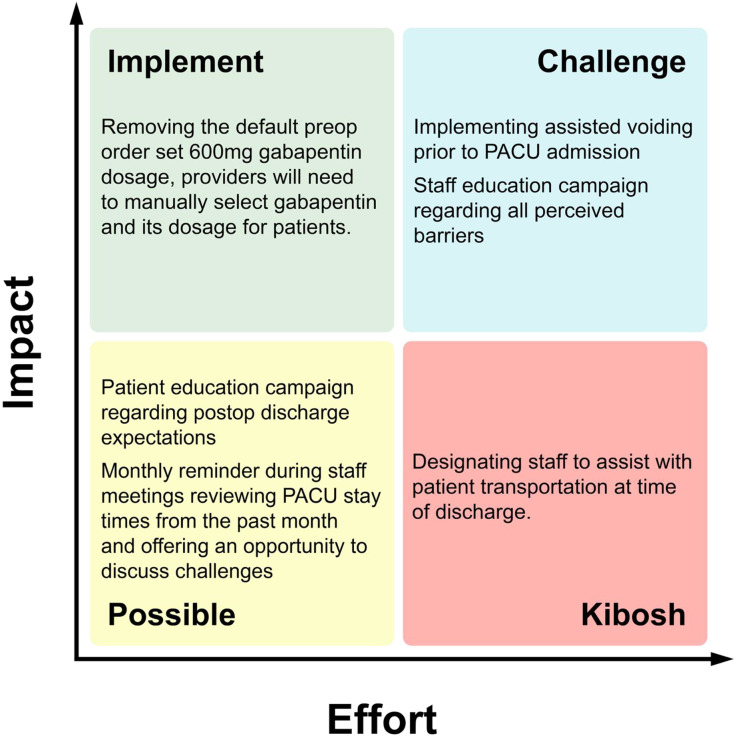
PICK chart identifying potential interventions proposed by the study team.

Data from 2021 was further analyzed to characterize PACU length of stay in the adult population undergoing these procedures, particularly those receiving minimally invasive or robotic surgeries. Data was stratified to evaluate PACU stay times across patient age groups, race, procedure length, and BMI. Medication records were reviewed to assess the prevalence of pre-operative gabapentin administration among patients undergoing outpatient benign gyn surgery; gabapentin was added to the perioperative EMR order set at this site in 2016 to reduce opioid prescribing. Building on this analysis, a PICK chart was then assembled to evaluate proposed interventions based on their estimated impact and required effort (Fig 2). Interventions were plotted on the effort-impact matrix to facilitate consensus on implementation priorities. The intervention, removal of the default pre-op gabapentin order set, was identified as high-impact and low-effort and was therefore selected for implementation.

### Intervention implementation

Given the described project formation, it was hypothesized that a major contributing factor to prolonged PACU stay times for benign gyn surgical procedures was overuse of gabapentin in the perioperative setting. To test the hypothesis and address gabapentin overuse, the study removed the default pre-op order set of 600 mg gabapentin after careful consideration with the anesthesia and surgical team. After the modification, surgical providers had to manually select if gabapentin is required and what dose for each individual patient undergoing outpatient benign gyn surgery. In addition to changing the order set, the experiment included a department-wide email notification of the new order set protocol.

### Data collection and analysis

Case records were extracted from Epic for adult patients (≥18 years) undergoing outpatient benign gynecologic surgeries at a single tertiary-care hospital between 01/03/2022 and 30/06/2024. Eligible procedures included a heterogeneous mix of benign gynecologic surgeries designated as outpatient at the time of scheduling, including laparoscopic, robotic-assisted, minimally invasive, and open approaches.

Inpatient surgeries were excluded, as were procedures initially intended to be outpatient that subsequently required inpatient admission due to perioperative or postoperative complications. Malignant, oncologic, and emergency procedures were not included.

Patient-level variables extracted included age, race, ethnicity, preferred language, sex, marital status, and body mass index (BMI). Clinical variables included preoperative and postoperative pain scores, post-anesthesia care unit (PACU) length of stay (minutes), total length of stay, and preoperative gabapentin administration. Data were accessed for research purposes on 25/06/2025.

Data integrity was verified by the study team upon extraction. An initial preliminary analysis was performed four months after project launch to ensure data fidelity. Pre-intervention data included cases from one year prior to the implementation period, and post-intervention data included cases from one month following the intervention through the subsequent year. For race, PACU stay times were compared pre- and post-intervention using a one-way ANOVA with a Brown-Forsythe and Welch’s correction. All other variables were analyzed by unpaired and Welch’s t-tests. PACU stay times were compared pre- and post-intervention using unpaired and Welch’s t-tests. GraphPad Prism was used for statistical analysis.

The primary outcome was average PACU length of stay. Secondary outcomes included changes in gabapentin prescribing practices, postoperative pain scores, and the influence of demographic and clinical characteristics on PACU duration. This quality improvement project underwent expedited review by the Institutional Review Board.

No changes were made to PACU pain management protocols or anesthetic techniques beyond changing order sets fir gabapentin during the study period; procedure-appropriate regional or local anesthetic blocks were used at the discretion of the anesthesiology team both before and after the intervention.

### Ethics statement

This study was approved by the University of California, San Francisco (UCSF) Institutional Review Board, Laurel Heights Committee (IRB #24–42687; Approval Date: February 6, 2025). The IRB determined the study to be minimal risk and granted a waiver of individual informed consent, as the research involved retrospective review of data collected for non-research purposes. The requirement for individual Research HIPAA Authorization was also waived, as the study met regulatory criteria for protection of privacy and confidentiality.

## Results

**Fig 3:**
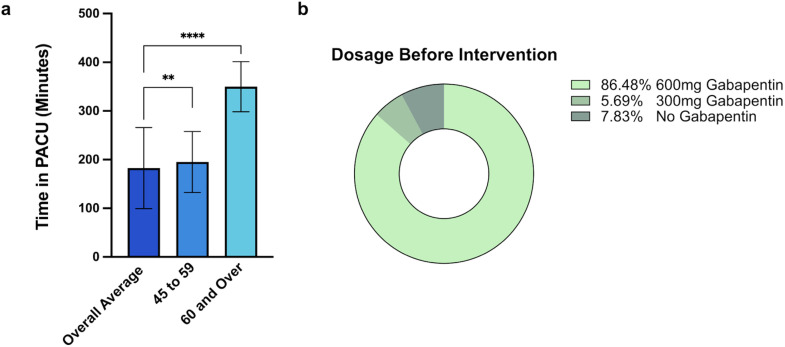
Average PACU times and gabapentin use from 2019-2021. **(a)** Overall average PACU stay time of 182.66 minutes, 194.86 minutes in patients aged 45-59 (p = 0.0054), and 349.92 minutes in patients aged 60+ (p < 0.0001). **(b)** Overall, in patients undergoing benign gynecological surgery, 86.48% received 600 mg, 5.69% received 300 mg, and 7.83% received no gabapentin.

Data from 2021 shows that outpatient benign gynecologic surgery PACU stays in the MB adult population averages 183 minutes. Data was analyzed for disparities in PACU times based on different sociodemographic markers and clinical factors including race/ethnicity, BMI, procedure length, procedure type, and age (Table 1). There was no difference found in PACU time based on race or BMI. There was a positive correlation between procedure length and PACU stay but it was not statistically significant (S1 Fig). After stratification of the data set by age, it was found that patients over the ages of 45 and 60 experienced significantly longer PACU stays (Fig 3a). Patients 60 years and older had significantly longer PACU stay times than patients 45–59 years of age (350 vs 195 minutes, p = 0.0054). Prior to intervention, over 90% of patients received gabapentin.

**Table 1:** Summary demographics of analyzed patient population.

Variable	Pre-Intervention	Post Intervention
Age (years) 18-44 45-59 60+ Race/Ethnicity African American Asian Hispanic White Other BMI <24.9 25+ Procedure Length < 2 Hours >2 Hours Procedure Type Minimally Invasive Open Robotic	69.8% (n = 196) 25.0% (n = 70) 5.3% (n = 15) 16.0% (n = 45) 18.9% (n = 53) 18.2% (n = 51) 37.7% (n = 106) 9.2% (n = 26) 50.9% (n = 143) 49.1% (n = 138) 62.6% (n = 176) 37.4% (n = 105) 23.9% (n = 67) 64.5% (n = 181) 11.6% (n = 33)	69.8% (n = 400) 22.3% (n = 128) 7.9% (n = 45) 10.6% (n = 61) 16.8% (n = 96) 15.7% (n = 90) 54.3% (n = 311) 2.6% (n = 15) 40.5% (n = 232) 59.5% (n = 341) 53.9% (n = 309) 46.1% (n = 264) 28.4% (n = 163) 42.6% (n = 244) 29.0% (n = 166)

**Fig 4:**
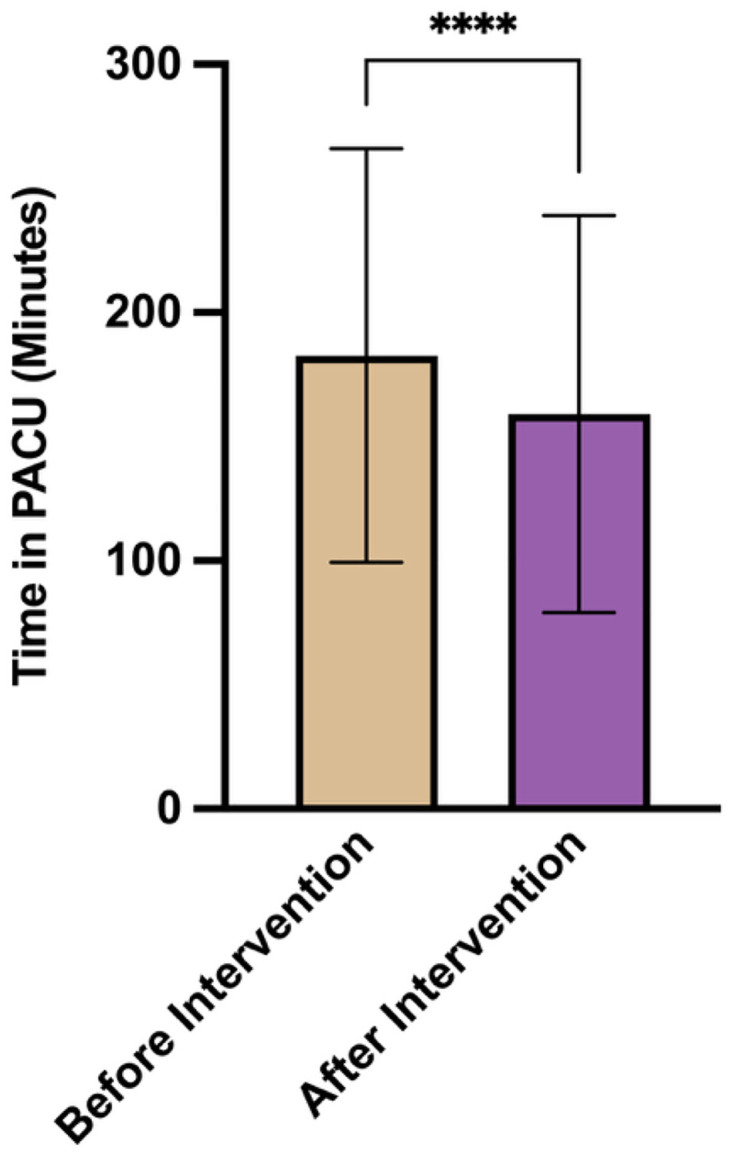
PACU stay times before and after intervention. PACU stay times before the proposed intervention averaged at 182.67 minutes and 159.16 minutes 1 year post implementation of the intervention (p < 0.0001).

**Fig 5:**
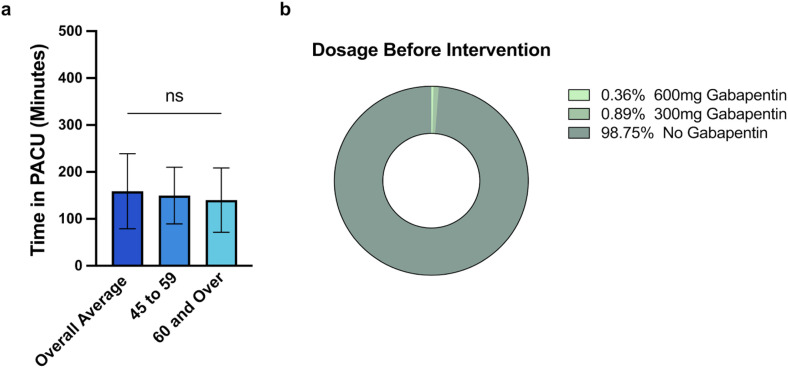
Average PACU times and gabapentin 1-year post-intervention. **(a)** Overall, in patients undergoing benign gynecological surgery, 0.6% received 600 mg, 0.89% received 300 mg, and 98.75% received no gabapentin. **(b)** Overall average PACU stay time of 159.16 minutes, 149.94 minutes in patients aged 45-59 (p = 0.1446), and 140.09 minutes in patients aged 60+ (p = 0.0818).

One year after intervention implementation, the average PACU stay time for benign gyn surgeries was reduced from an average of ~183 to ~159 minutes marking a 12.6% reduction in PACU stay time (Fig 4). To further validate that this reduction was in part due to the intervention, data was analyzed to evaluate gabapentin use and saw that gabapentin use decreased from 92.17% of all cases to 1.25% after the intervention (Fig 5a). PACU times were once again analyzed using the same sociodemographic markers and there were no observed trends or statistical differences in PACU stay times amongst groups. Importantly, the previously observed positive correlation between age and PACU stay time was attenuated following the intervention (Fig 5b). These results suggest that gabapentin may have played an important role in the prolonged PACU stay times, and its removal negated the observed trend.

**Fig 6:**
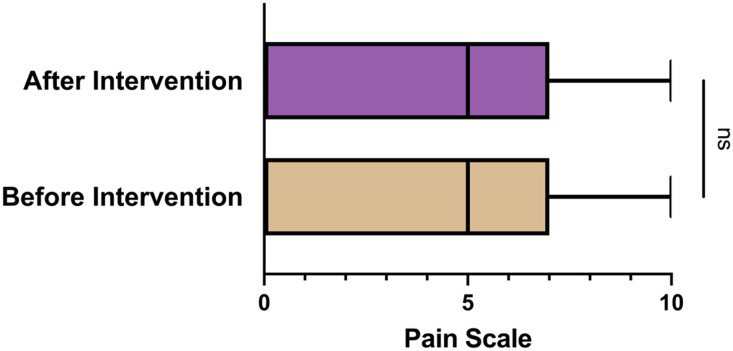
Patient pain scales before and after intervention. Patient pain scales before the proposed intervention averaged at 4.35. One-year post implementation of the intervention, patient pain scales were reported at an average of 4.36 (p = 0.9621).

To assess the impact of reduced perioperative gabapentin use on patient satisfaction and safety, postoperative pain scores were compared before and after the implementation. Notably, pain scores remained consistent across both time periods, suggesting that the significant decrease in gabapentin administration did not adversely affect pain control in this population, despite its routine use for perioperative pain management (Fig 6).

## Discussion

This quality improvement project aimed to address prolonged PACU stay times among patients undergoing outpatient benign gynecologic surgery at a high-volume academic medical center facing bed availability challenges. Initial data review confirmed that this surgical population had the longest average PACU stays across all departments, far exceeding the literature-based target of 120 minutes [4].

After the study team identified primary barriers to timely PACU discharge, subsequent chart review highlighted that a majority of patients, including older adults, routinely received 600 mg of gabapentin as part of a standardized pre-operative order set. Gabapentin was originally included to reduce perioperative opioid use as suggested by several randomized control studies and a major 2016 meta-analysis [5]. Preoperative use of gabapentin has also been suggested to reduce post-operative nausea and vomiting [6]. However, new research suggests that gabapentin may increase PACU stay times due to excessive sedation [7]. Additionally, the preoperative gabapentin is associated with increased complications for older patients including increased risk for delirium [8]. To test if gabapentin overuse was a contributing factor to prolonged PACU stays, the default 600 mg gabapentin pre-operative order set was removed, and instead clinicians had to manually select gabapentin and its dosage.

Post-intervention analysis demonstrated a substantial reduction in PACU stay times, from an average of 183–159 minutes, representing a 12.6% improvement. This reduction coincided with a marked decrease in gabapentin use, from over 90% of cases to just over 1%. This aligns with existing research that suggests preoperative gabapentin use can lead to longer discharge times in gynecological procedures [9]. Notably, the previously observed age-related disparity in PACU times was attenuated, supporting the hypothesis that standard gabapentin dosing disproportionately affected older adults. Crucially, the intervention did not negatively impact patient pain control, suggesting that reducing or eliminating routine gabapentin use in this context does not compromise analgesia. This finding demonstrates both the efficacy and safety of this intervention.

While the primary goal of the project was to reduce PACU stay times, the success of the intervention also highlights the importance of continually reassessing standardized order sets, particularly in populations with known vulnerabilities to specific medications. In this case, removing a one-size-fits-all approach to gabapentin prescribing not only improved operational efficiency but also promoted more individualized and age-conscious care. These principles can be expanded to other perioperative settings to improve hospital efficiency and patient safety.

## Conclusions

A quality improvement intervention was implemented to address the prolonged PACU stay times of outpatient benign gynecological surgery at a single medical center. By changing the standardized pre-op order set of 600 mg gabapentin to manual selection of gabapentin dosing, PACU stay times decreased significantly. Notably, disparities in PACU stay times between older (>60 years of age) and younger patients was attenuated. This project provides a roadmap for similar interventions across other surgical specialties and demonstrates the importance of continued assessment of standardized order sets to promote quality, individualized, and age-conscious care.

## Supporting information

S1 Fig Stratified analysis of PACU stay times by race, procedure length, and BMI. (a) PACU durations showed no statistically significant differences across racial groups (Asian, African American, White, and Other). (b) A positive correlation was observed between procedure length and PACU stay time; procedures lasting over 2 hours were associated with longer PACU stays (183.85 minutes) compared to those under 2 hours (168.56 minutes). (c) Patients with a BMI ≤ 24.9 had an average PACU stay of 177.04 minutes, while those with a BMI > 25 had an average stay of 188.48 minutes. Statistical significance was assessed as indicated; none of the observed associations reached conventional thresholds for significance.
(DOCX)

S1 File Data Availability De-identified raw data. All relevant data are within the manuscript and its Supporting Information files. De-identified raw data used to generate all figures in the manuscript are provided as a Supporting Information Excel file.
(XLSX)
