# Supplementary figures and images for: Modification of pre-operative order set to reduce PACU stay times for outpatient benign gynecological surgery
Source: PLoS One. 2026 Jun 10;21(6):e0336194. doi: 10.1371/journal.pone.0336194 (PMC13252802; doi:10.1371/journal.pone.0336194)

Supplemental Figure


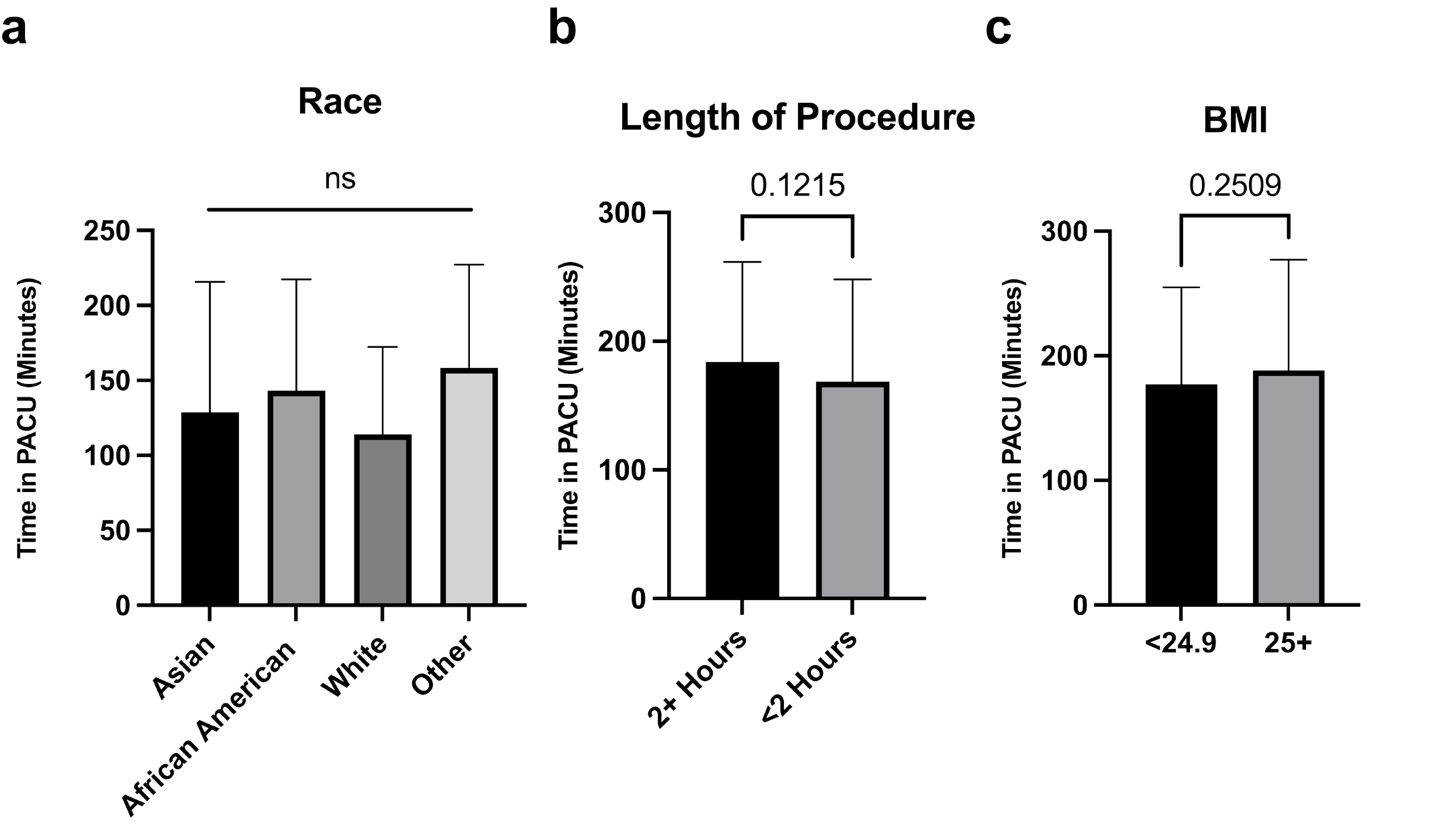

Supplement: S1 Fig — (a) PACU durations showed no statistically significant differences across racial groups (Asian, African American, White, and Other). (b) A positive correlation was observed between procedure length and PACU stay time; procedures lasting over 2 hours were associated with longer PACU stays (183.85 minutes) compared to those under 2 hours (168.56 minutes). (c) Patients with a BMI ≤ 24.9 had an average PACU stay of 177.04 minutes, while those with a BMI > 25 had an average stay of 188.48 minutes. Statistical significance was assessed as indicated; none of the observed associations reached conventional thresholds for significance. (DOCX) [file pone.0336194.s001.docx]
